# A Comparative Analysis of In Vitro Toxicity of Synthetic Zeolites on IMR-90 Human Lung Fibroblast Cells

**DOI:** 10.3390/molecules26113194

**Published:** 2021-05-26

**Authors:** Seung-Hye Yu, Manjesh Kumar, Il Won Kim, Jeffrey D. Rimer, Tae-Jung Kim

**Affiliations:** 1 Department of Chemical Engineering, Soongsil University, Seoul 06978, Korea; koreaush@naver.com; 2 Department of Chemical Engineering, Indian Institute of Technology Delhi, Delhi 110016, India; kr.manjesh@gmail.com; 3 Department of Chemical and Biomolecular Engineering, University of Houston, Houston, TX 77004, USA; 4 Department of Hospital Pathology, College of Medicine, The Catholic University of Korea, Seoul 06591, Korea

**Keywords:** zeolite, fibroblast, lung, IMR-90, cytotoxicity, glutathione

## Abstract

Broad industrial application of zeolites increases the opportunity of inhalation. However, the potential impact of different types and compositions of zeolite on cytotoxicity is still unknown. Four types of synthetic zeolites have been prepared for assessing the effect on lung fibroblast: two zeolite L (LTL-R and LTL-D), ZSM-5 (MFI-S), and faujasite (FAU-S). The cytotoxicity of zeolites on human lung fibroblast (IMR-90) was assessed using WST1 cell proliferation assay, mitochondrial function, membrane leakage of lactate dehydrogenase, reduced glutathione levels, and mitochondrial membrane potential were assessed under control. Intracellular changes were examined using transmission electron microscopy (TEM). Toxicity-related gene expressions were evaluated by PCR array. The result showed significantly higher toxicity in IMR-90 cells with FAU-S than LTL-R, LTL-D and MFI-S exposure. TEM showed FAU-S, spheroidal zeolite with a low Si/Al ratio, was readily internalized forming numerous phagosomes in IMR-90 cells, while the largest and disc-shaped zeolites showed the lowest toxicity and were located in submembranous phagosomes in IMR-90 cells. Differential expression of TNF related genes was detected using PCR arrays and confirmed using qRT-PCR analysis of selected genes. Collectively, the exposure of different zeolites shows different toxicity on IMR-90 cells.

## 1. Introduction

Zeolites are crystalline aluminosilicates with disparate pore dimensions and networks of channels or cages that can be synthesized with a range of particle size and morphology. They are widely used as catalysts and adsorbents in industry [1]. Zeolites also can be synthesized in various forms and compositions to improve their properties for diverse commercial applications [2]. Despite experimental studies on zeolite-related diseases, the identification of a single physicochemical feature responsible for adverse responses is lacking. The cytotoxic effects of synthetic zeolites, depending on their shape, composition, size [3], surface charge [4], corona proteins in suspension [5], and cellular internalization [6] have been demonstrated. Water adsorption and ion-exchange capabilities of zeolites, however, have led to their use in pharmaceutical applications as a contrast medium in diagnostic methods, thereby emphasizing the dichotomy of microporous materials [7]. In many cases, zeolites enter the cell after binding to the receptor target. Once bound, several factors such as geometric shape and composition can dictate the behavior of zeolites at the cell-particle interface [3,8], and will typically enter the cell via receptor-mediated endocytosis forming phagosome; however, the behavior of particles in phagosome remains largely unknown depending on size, shape, ligand density and intracellular localization [9,10]. The increasing demand for zeolites in commercial applications has motivated studies on the toxicological effect of zeolite inhalation [5,11,12]. In general, inhaled particles can give rise to pulmonary interstitial fibrosis, a pathologic form of occupational lung disease [13,14,15] or even lung cancer [16,17]. The interstitial accumulation of inhaled particles with surrounding fibroblast proliferation is frequently observed during pathological examination of lung tissue from the patients with the history of the particle exposure [18]. The association of the bioactivity and toxicity with fibroblast by the physicochemical properties of particles have been investigated [4]. Fibroblasts are mainly responsible for excess deposition of extracellular matrix and for a microenvironment for pulmonary fibrosis [19] and lung cancer [20]. Our previous study revealed the exposure of fibroblast to asbestos inducing differential expression of cytotoxicity related mRNA profiles according to different asbestos [21] and the medium from fibroblast cultured with asbestos enhancing metastatic potential of lung cancer cell line [22]. Recent studies regarding the disease related to environmental or occupational particles are laying emphasis on low dose continuous exposure to particles, because such models are compatible with particle-exposed patients [23]. Due to largely unknown effect of zeolite exposure on lung fibroblast, we primarily aimed to evaluate the exposure effects of various synthesized zeolite particles to lung fibroblasts. As far as we know, this is the first study assessing toxicity of zeolites in IMR-90 lung fibroblasts.

## 2. Results

### 2.1. Physicochemical Properties of Zeolites

**Figure 1:**
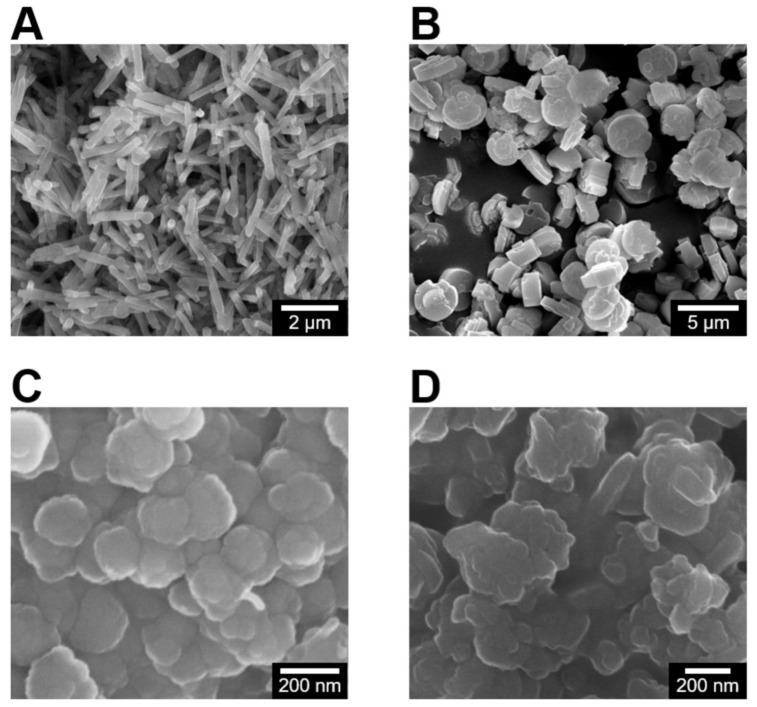
Scanning electron micrographs of the following as synthesized zeolite particles: (**A**) LTL-R, (**B**) LTL-D, (**C**) MFI-S, and (**D**) FAU-S.

**Figure 2:**
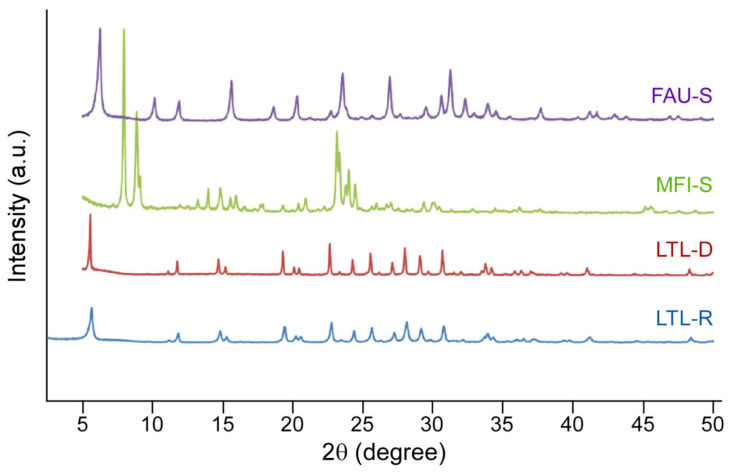
Powder X-ray diffraction patterns of the following as synthesized zeolite samples: FAU-S (purple), MFI-S (green), LTL-D (red), and LTL-R (blue). The patterns are arbitrarily offset along the y-axis for visual clarity.

The physical properties of zeolite crystals, such as particle size distribution, shape, and surface charge (or zeta potential), are measured. Scanning electron micrographs of zeolite particles demonstrated that the synthesized materials exhibit fairly uniform size and shape. The two zeolites with a LTL framework type, labelled LTL-R (Figure 1A) and LTL-D (Figure 1B), have identical compositions (Si/Al 3) and a crystal structure comprised of one-dimensional large pores (diameter 0.7 nm) with interconnected 12-, 8-, 6-, and 4-membered rings. LTL-R has dimensions in the nanoscale for its diameter, whereas LTL-D particles are microns in diameter with nanoscale surface variation. In addition, a distinct difference in morphology between LTL-R and LTL-D was achieved by controlling the anisotropic growth rate with zeolite growth modifiers [24]. Rod-shaped (LTL-R) and disc-shaped (LTL-D) crystals were prepared in the presence of PDDAC and 1-butanol, respectively, [25]. The MFI-S zeolite (Figure 1C), which has a MFI framework, is comprised of three-dimensional medium pores (diameter 0.55 nm) with interconnected pentasil chains. The morphology of MFI-S is spheroidal with an average diameter of 200 nm. The FAU-S zeolite (Figure 1D) has a faujasite (FAU) framework consisting of interconnected sodalite cages and double-6-membered rings to form three-dimensional large pores. The morphology of FAU-S is spheroidal with sizes in the range of 150 to 200 nm. Elemental analysis revealed the chemical composition (notably the Si/Al ratio) of MFI-S and FAU-S to be approximately 50 and 1.5, respectively. The framework structure of each zeolite was confirmed by powder XRD (Figure 2) and comparisons to reference patterns reported in the International Zeolite Association Structure Database [26]. The charge of the particle surface was quantified by zeta potential measurements. As shown in Table 1, all four zeolite samples suspended in DI water exhibit highly negative surface charge; however, the magnitude of zeta potentials decreased for each zeolite sample in contact with DPBS, serum-free EMEM (EMEM-N), and EMEM-F (EMEM supplemented with 10% FBS). In particular, the values in EMEM-F were significantly reduced to nearly −10 mV for all four zeolite samples: −10.5 ± 0.4 mV (LTL-R); −9.3 ± 0.5 mV (LTL-D); −10.3 ± 0.2 mV (MFI-S); −11.8 ± 0.6 mV (FAU-S). The changes in zeta potential in various zeolite particles indicated that the surrounding ions and medium (i.e., various biomolecules) alter their surface properties.

### 2.2. Real-Time Cell Response of IMR-90 to Zeolite Particles

**Figure 3:**
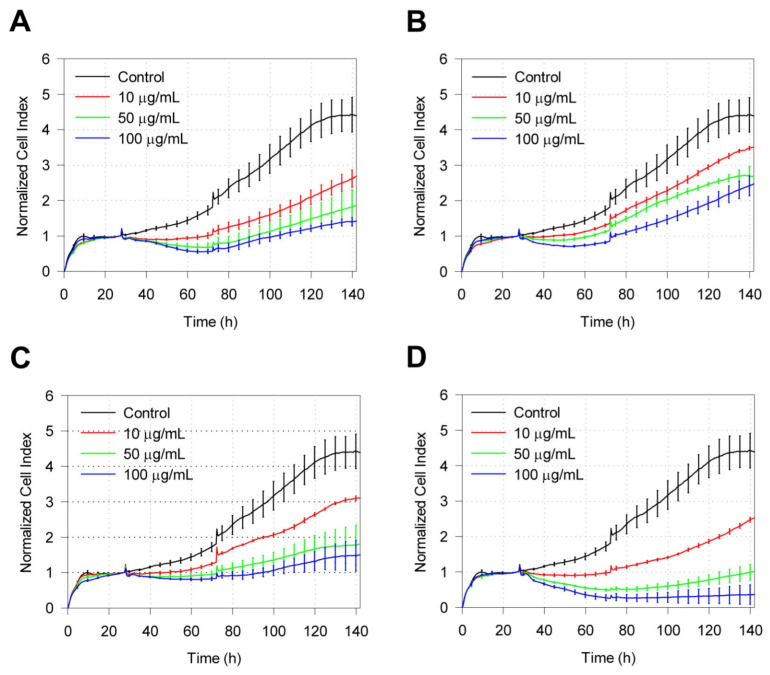
Dynamic assessment of the viability of IMR-90 cells exposed to zeolite particles. Exposing IMR-90 cells to different concentrations of zeolite particles (10, 50, and 100 μg/mL). The CI values were normalized at the time of the zeolite treatment for the following crystal structures: (**A**) LTL-R, (**B**) LTL-D, (**C**) MFI-S, and (**D**) FAU-S. Control cells cultured in zeolite-free media were run parallel to treatment group. Experiments were performed in quadruplet and presented as mean ± SD (n = 3). CI, cell index.

Real-time cell analysis sensing profiles (normalized CI) were generated by exposing IMR-90 cells to different concentrations of zeolite particles. All types of zeolite exposure to IMR-90 showed a time- and dose-dependent inhibitory effect toward the growth of IMR-90 cells. For all zeolites, Normalized CI was the lowest for 72 h exposure. When exposed to 50 and 100 μg/mL of synthesized zeolites, inhibitory effects on IMR-90 for samples LTL-R (Figure 3A) and MFI-S (Figure 3C) showed similar patterns, while the weakest and strongest inhibitory effects were observed for LTL-D (Figure 3B) and FAU-S (Figure 3D), respectively.

### 2.3. Cell Viability Study

**Figure 4:**
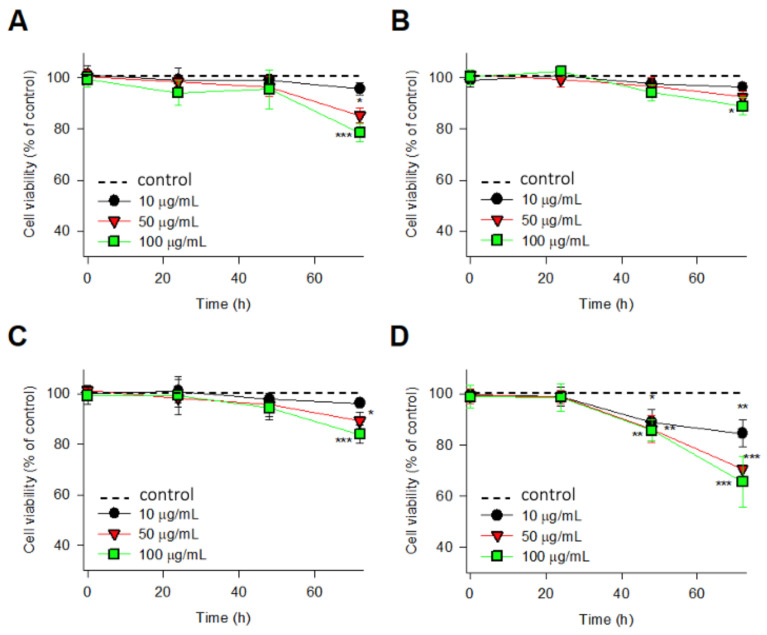
Cell viability of IMR-90 cells in the culture treated with 10, 50, and 100 μg/mL of (**A**) LTL-R, (**B**) LTL-D, (**C**) MFI-S, and (**D**) FAU-S for 72 h. All data are represented as the mean ± SD compared to the control. Symbols *, **, and *** indicate the statistically significant difference with *p* values <0.05, <0.01, and <0.001, respectively.

IMR-90 cells were incubated with different concentrations (0, 10, 50 and 100 μg/mL) of zeolites and viability was determined 2, 24, 48, and 72 h by water soluble tetrazolium salt WST assay after treatment. As shown in Figure 4A–C, treatment of IMR-90 cells with zeolite LTL-R, LTL-D, and MFI-S did not significantly alter cell viability (%) relative to the control until 48 h. When the exposure time of zeolite particles is prolonged to 72 h, the reduced viability of zeolite LTL-R, MFI-S and FAU-S became more obvious (*p* < 0.05) beyond 50 μg/mL. Zeolite LTL-D had the highest viability and FAU-S had the lowest viability against IMR-90 cells after 72 h exposure. Even with prolonged exposure time (72 h), zeolite LTL-D only displayed a little reduced viability at high concentration (Figure 4B).

### 2.4. Cytotoxicity Study

**Figure 5:**
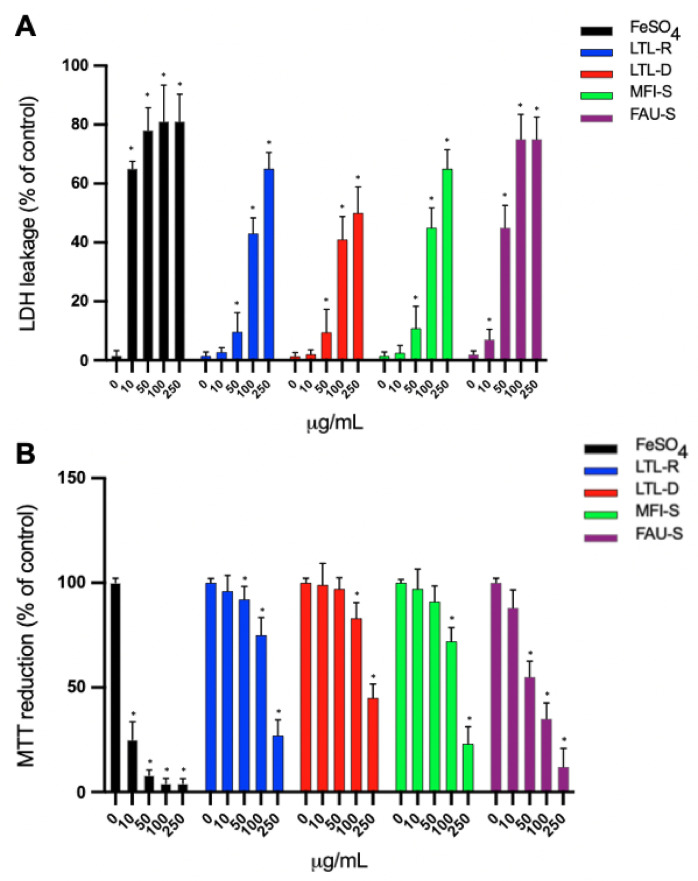
Cytotoxic effect of zeolites on lung fibroblast IMR-90 cells. Cells were treated with different concentration of zeolites for 72 h. (**A**) LDH leakage in medium. The percent of LDH activity was calculated by dividing the amount of acivity in the medium by the total activity (medium and cell lysate). Controls were cultured in zeolite free media were run in parallel to the treatment group. (**B**) Effect of zeolite on mitochondrial function determined by MTT reduction in IMR-90 human lung fibroblasts. The cells were incubated for 72 h with various concentrations (0, 10, 50, 100, 250 μg/mL) of zeolite LTL-R, LTL-D, MFI-S and FAU-S. Ferrous sulfate (FeSO${}_{4}$) was used as positive control. The results are presented as mean values ± SD versus unexposed cells to zeolites. The data are expressed as mean ± SD of three independent experiments. * indicates a statistically significant differences compared to control.

The results demonstrated that exposure to FAU-S resulted in a concentration dependent increase in LDH leakage and exhibited a significant cytotoxicity at 10 μg/mL (*p* < 0.05) (Figure 5A). Ferrous sulphate (FeSO${}_{4}$) was used as a positive control [11,27]. It was obeserved that there is a significant difference between FAU-S and the other zeolites. The results for LDH leakage for LTL-R, LTL-D and MFI-S zeolites exposure did not show definite cytotoxicity up to 50 μg/mL. Based on LDH results, FAU-S was the most toxic when compared with the other zeolites. The result of MTT assays also showed FAU-S having a significant toxicity on IMR-90 cells at 10 μg/mL (*p* < 0.05) (Figure 5B). In contrast, the other zeolites did not produce a significant increase in cytotoxicity at the 10 μg/mL of concentration. FAU-S, spheroidal zeolite with a low Si/Al ratio, showed highest cytotoxicity, while the largest and disc-shaped zeolites had the lowest cytotoxicity. The EC${}_{50}$ values of LDH leakage and MTT are presented in Table 2.

### 2.5. Mitochondrial Membrane Function Disruption

**Figure 6:**
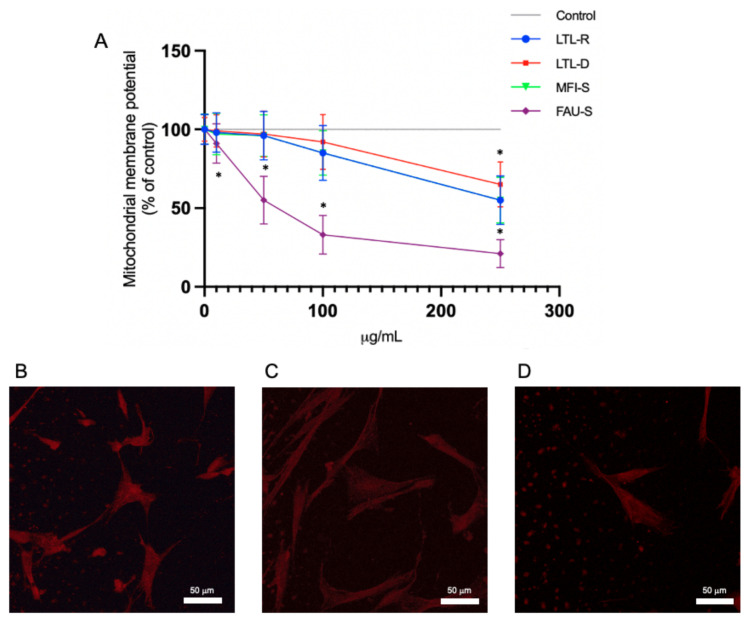
(**A**) Effect of zeolites on mitochondrial membrane potential in lung fibroblast IMR-90 cells. Cells were exposed to different concentration of zeolites for 72 h. The data are expressed as mean ± SD of three independent experiments. * indicate statistically significant differences compared to control. Morphologic chracterization of mitochondrial disruption by MitoTracker Red staining: (**B**) control, (**C**) cells with 50 μg/mL FAU-S, and (**D**) cells with 100 μg/mL FAU-S were visualized using cofocal laser microscopy. Scale bar: 50 μm.

The effect of zeolites on mitochondrial membrane potential (MMP) was evaluated in IMR-90 cell (Figure 6A–D). Cells were exposed to 0, 10, 50, 100 and 250 μg/mL zeolites for 72 h and immediately assayed for rhodamine 123 uptake. The results indicated that there was a significant decrease of MMP over 50 μg/mL FAU-S (Figure 6A). The morphological assessment of mitochondrial membrane disruption was observed under confocal laser microscope stained with MitoTracker Red. The brightness of the fluorescent intensity was reduced in cells exposed to FAU-S at more than 10 μg/mL that indicating a significant reduction of mitochondrial membrane potential (Figure 6B–D). Actinomycin (5 μg/mL) was used as a positive control reagent to trigger apoptosis (data not shown).

### 2.6. Total Glutathione Levels

**Figure 7:**
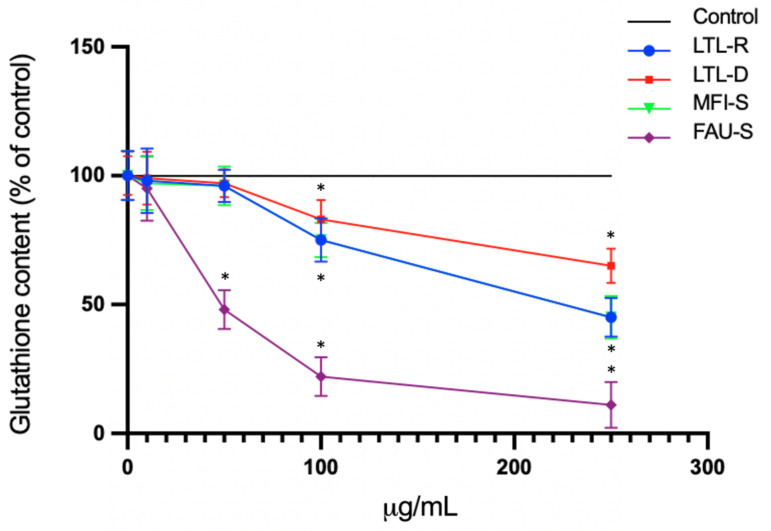
Effect of zeolites on GSH levels in lung fibroblast (IMR-90 cells). Cells were treated with zeolite LTL-R, LTL-D, MFI-S, FAU-S for 72 h. At the end of the exposure, cells were washed with PBS, and GSH (control: 61 ± 5 nmol/mg protein). Control cells cultured in zeolite-free media were run in parallel to treatment groups. The data are expressed as mean ± SD of three independent experiments. * indicates a statistically significant difference compared to controls (*p* < 0.05).

To assess the involvement of oxidative stress in particle mediated inflammation, the amount of total glutathione (both oxidised (GSSG) and reduced GSH, with the GSSG converted to GSH) after 72 h treatment with different concentration (0, 10, 50, 100, 250 μg/mL) of zeolites were measured. A significant depletion of GSH with FAU-S was observed at 50 μg/mL relative to controls (Figure 7). Control cells cultured in zeolite-free media were run in parallel to treatment groups.

### 2.7. Cellular Internalization of Zeolite Particles

**Figure 8:**
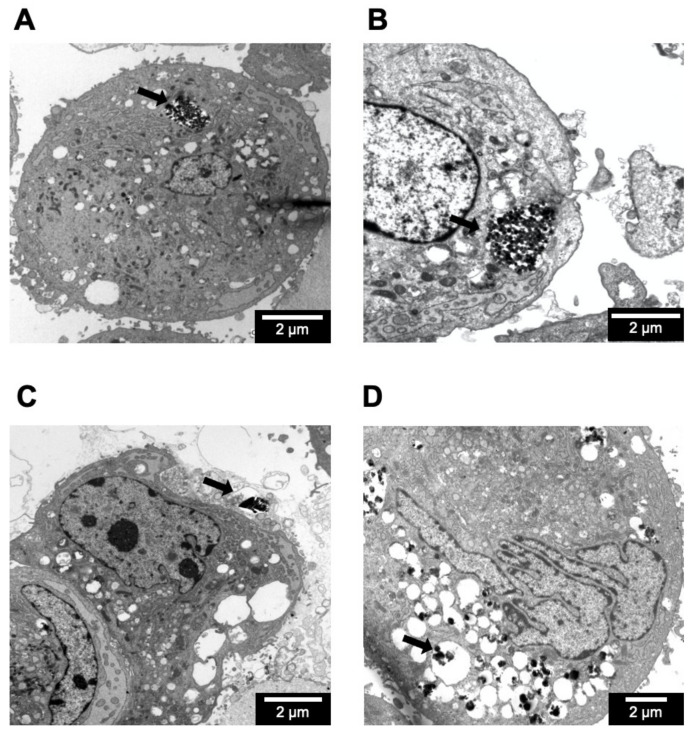
Ultrastructural features of zeolite particle uptake by IMR-90 cells. The transmission electron micrographs show thin sections with zeolite particle-exposed IMR-90 cells. The cells were cultured in the presence of (**A**) 50 μg/mL LTL-R, (**B**) 50 μg/mL MFI-S, (**C**) 50 μg/mL LTL-D, and (**D**) 50 μg/mL FAU-S for 72 h. The zeolite particles (arrow) were located as (**A**,**B**) aggregates in intracytoplasmic phagosome, (**C**) aggregates in submembranous phagosome, and (**D**) small aggregates in numerous cytoplasmic phagosomes.

To confirm that zeolites were internalized by IMR-90 cells and to determine the intracellular distribution of zeolites in the IMR-90 cells, ultrastructural examination using transmission electron microscopy for 72 h-incubation of IMR-90 with 50 μg/mL zeolite particles was performed (Figure 8). Zeolite particles were found as aggregates within intracytoplasmic or submembranous phagosome in IMR-90 cells. Ingested particles were also found as aggregates in intracytoplasmic phagosome in IMR-90 cells with LRL-R (Figure 8A) and MFI-S (Figure 8B), whereas IMR-90 cells with LTL-D revealed zeolite particle aggregates invaginated in submembranous location (Figure 8C). IMR-90 with FAU-S (Figure 8D) revealed numerous swollen phagosomes containing particle aggregates. The location and distribution of particles internalized in IMR-90 cells was different for each zeolite. The distribution and location of FAU-S zeolite were distinctly different from LTL-R, LTL-D, and MFI-S zeolites.

### 2.8. Toxicity Related Gene Expression

**Figure 9:**
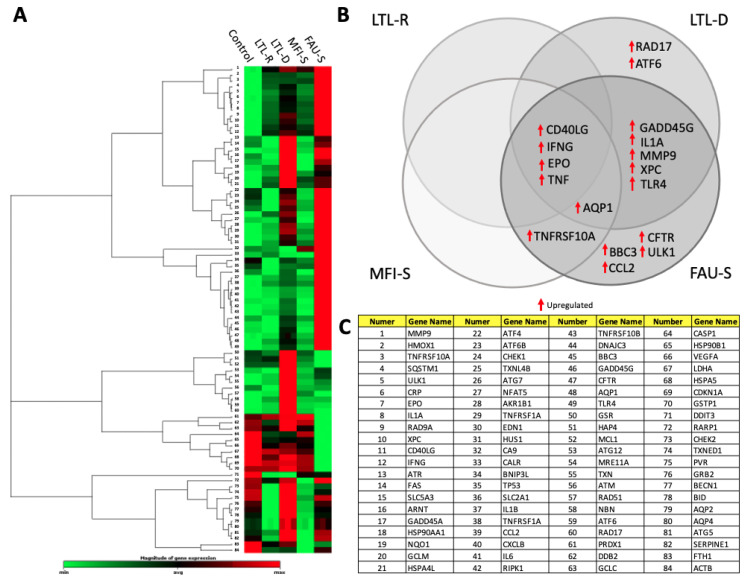
Gene expression profiles in IMR-90 cells after exposure to 50 μg/mL of zeolites (**A**) Clustergram of gene expression exposed to 50 μg/mL of LTL-R, LTL-D, MFI-S and FAU-S using PCR arrays. (**B**) Significantly altered gene expression with more than 2-fold change is shown in Venn diagrams organized by zeolite type. (**C**) Gene list of PCR array panel.

IMR-90 cells were evaluated using PCR array containing key genes and pathways associated with stress and toxicity (Figure 9). RNA was isolated from IMR-90 after 72 h exposure to 50 μg/mL LTL-R, LTL-D, MFI-S and FAU-S, then used for quantitative analysis of gene expression. Genes significantly changed (higher than a 2-fold change in expression) in at least one treatment group are highlighted using a clustergram to show changes relative to untreated IMR-90 cells (control) (Figure 9A). The most significantly altered genes showed that diffential expression of their genes is exposure specific (Figure 9B,C). Several highly upregulated genes, including CD40LG, TNF, IFNG and EPO, were induced by all types of zeolites but were highest in cells with FAU-S exposure. Because both CD40LG and TNF are of the TNF receptor family, dose-dependent expression of CD40LG and TNF was confirmed using qRT-PCR (Table 3). Expression of CD40LG and TNF-alpha was increased by 27 h-exposure to zeolites. Statistically significant increases of CD40LG and TNF-alpha were observed in IMR-90 cells after 72 h-exposure to MFI-S and FAU-S.

## 3. Discussion

Zeolites are silicate or aluminosilicate nanomaterials with well-defined pore networks and inhalation of these particles can lead to local cellular inflammation, cytokine responses, silicosis and increased rates of lung cancer. Even with recent advances in the field, the precise mechanism of toxicity is poorly understood [28]. The role of fibroblast in pulmonary fibrosis [21] and carcinogenesis [22] related to inhaled particles is increasingly noted. We synthesized four types of industrially relevant aluminosilicate zeolites for assessing their cytotoxic effect on human lung fibroblast. Zeolite LTL is a hexagonal crystal (space group; P6/mmm) [29] with one-dimension channels oriented in the c-direction [30]. The MFI framework structure is orthorhombic (space group; Pnma) with a three-dimensional network of interconnecting channels along with the a- and b-axes. The elemental composition of MFI (Si/Al = 50) is different from LTL (Si/Al = 3) and FAU (Si/Al = 1.5) [31]. The latter is a cubic crystal composed of interconnected cages separated by 12-membered rings windows. All types of zeolites showed growth inhibition on IMR-90. Among them, FAU-S showed the strongest and LTL-D showed the weakest inhibitory effect on the growth of IMR-90 cells. Both LTL-R and LTL-D have different zeta potentials in deionized water depending on their crystal planes. Due to differences of hydroxyl deonized (DI) water on distinct crystallographic faces, LTL-D (4.2 OH groups/nm${}^{2}$ on faces) had a higher zeta potential than LTL-R (1.5 OH groups/nm${}^{2}$ on faces), in agreement with previous results [25]. The zeolite with the highest aluminum content, FAU-S, was more negatively charged in DI water than the zeolite particles with the least aluminum content, e.g., MFI-S [32]. However, in the biological environment, it is likely that biomolecules and ions are readily adsorbed on zeolite crystal surfaces and form a protein corona [33], thus reducing the zeta potential [11]. The surface charges of zeolite samples in various media, summarized in Table 1, confirmed that the zeta potential of the zeolite particles decreased in magnitude when the crystals were suspended in DPBS, cell culture media, and serum-containing cell culture media. We hypothesize the negatively charged cellular membrane enables lesser negatively charged particles to have a stronger affinity for the cell membrane, thus helping them to be internalized through the membrane. However, a former study reported an opposite effect where particles with a negative zeta potential were also readily internalized [34]. The uptake of negatively-charged particles putatively begins with adsorbing to specific binding sites with positive charges dispersed on the cell membrane [35,36]. Then, the particles are agglomerated by electrostatic repulsion between the negatively charged particles and the negative domains of the cell membrane. If proteins or other biomolecules adsorb on zeolite surfaces, this could promote particle aggregation as well. In the FBS-containing EMEM, the low zeta potential of particles allows them to overcome electric repulsion that further promotes particle clustering [37]. Transmission electron microscopy images for cellular ultrastructural examination proved the cellular uptake of zeolite particles exhibiting negative zeta potential in EMEM media containing FBS. The least uptake was exhibited by LTL-D exposure to IMR-90 cells showing as a form of aggregation in submembranous phagosome. Prior studies have reported that the particle size affects cytotoxicity. For example, small gold nanoparticles easily enter the cell nucleus, but larger nanoparticles (10 or 16 nm) can penetrate the cell membrane but are found only in the cytoplasm (Huo et al. 2014). In fibroblasts, epithelial cells, macrophages, and melanoma cells, nanoparticles of 15 nm in size were 60 times less toxic than 1.4 nm nanoparticles [38,39]. These observations are similar to our result that LTL-D, the largest particle, has less effect on the cells than the other zeolite particles. Since distinctive shapes with identical volumes had significant influences on the cellular internalization, the surface area of the disc shape is smaller than that of the rod shape with a high aspect ratio and can be attributed to the narrow contact area to interact with the cell membrane [8]. Real time cell analysis showed the effect of zeolite is dose and time dependent. We determined 72 h exposure for most experiments in our study, which is the time point when growth of IMR-90 cells were observed to be the most suppressed by zeolite exposure. Further study considering both time and dose factor is needed to generalize our theory. The FAU-S and MFI-S particles have an identical shape, but FAU-S has significantly higher aluminum content than MFI-S. Each aluminum within the framework of a zeolite is negatively charged and is compensated by a counterion; thus, higher aluminum indicates a greater ion-exchange capacity to potentially catalyze intracellular reactions, followed by cytotoxicity [3] and necrosis [40]. The higher cytotoxicity observed for FAU-S could be due to its low Si/Al ratio in comparison with MFI-S, which has a much higher Si/Al ratio. Furthermore, the finding of widely dispersed phagosome formation with the FAU-S exposure in IMR-90 is distinctly different from other zeolites and could be associated with the highest cytotoxicity of FAU-S. The formation of numerous phagosomes is regarded as a major initiation factor for necrotic cell death [41]. The phagosome containing particles in the cytoplasm trigger a series of events that interfere with mitochondrial function or activate the stress-mediated signaling cascade [42], and the adhesion molecules of the particles affect cytoskeleton remodeling and cell growth [43]. These series of actions eventually cause the cytotoxic events. It is noted that 50 μg/mL concentration of FAU-S depleted GSH significantly. There are remaining questions regarding how zeolite could induce toxicity. It is not known, for instance, how FAU-S depleted GSH levels, whether it binds directly to GSH or has an inhibitory mechanism on enzymes involving GSH synthesis. We demonstrated cellular internalization of zeolite particles and their relation to cytotoxicity, GSH depletion and their potential relationship to the physicochemical properties of particles (size, shape, composition) that often govern their value for industrial applications [44]. For example, rod-shaped zeolite nanoparticles can be used as microcapillary devices [45], whereas disc-shaped zeolite particles are useful as catalysts [46,47] and in in external devices to produce uniform single layers or films [48]. Zeolites with higher Si/Al ratio are used to recover 1-butanol [49] or selectively convert p-chlorophenol to formic acid [50] in wastewater. On the contrary, the lower Si/Al ratio is useful for removing arsenic in wastewater and the catalytic production of olefins in the petroleum industry [51]. We found increased gene expression of CD40L and TNF especially in FAU-S exposure to IMR-90 cells in comparison with the control. CD40L and TNF are both members of the TNF receptor family [52] and activate the classical NF-κB pathway [53] The relation between membrane bound TNF and cytolysis via juxtacrine inter-cell death signaling is observed [54]. However, our result for gene expression is preliminary and more studies of protein expression are needed. Another limit of our study is a lack of toxicity assessment. There are a few studies in the literature: according to the 2006 OECD Screening Information DataSet (SIDS) Initial Assessment Meeting, no toxic effects of crystaline, non-fibrous zeolites (zeolite A, P, X and Y) were observed after acute exposure to zeolites in use of rats and rabbits. In repeated dose inhalation study with cynomolgus monkeys, macrophage accumulation, bronchiolitis and alveolitis were found after 6 months with the lowest zeolite concentration of 1 mg/m${}^{3}$. However, no adverse effects were observed at high doses in chronic oral studies. Interestingly, in vivo clastogenesis studies showed no evidence of induction of chromosomal aberrations by either zeolite A or X. While, an study of zeolite X showed clear induction of chromosomal aberrations [55]. Intratracheal administration of ZSM-5 to rats caused the low level of toxicity that minor nonspecific pulmonary reactions were observed at 6 months after dosing [56]. Inhalation study with zeolite A revealed subchronic toxicity (11 weeks) in rats showing pneumonitis but showed no fibrotic reaction. On the other hand, chronic inhalation study (12 months) in hamster showed considerable incidence of death due to specific infection or moderate to extensive signs of respiratory disease [57]. One interesting finding in a toxicity study of mesoporous particles is that even though the particles show very little tissue toxicity, they do appear to invoke a systemic response and cause death of mice subjects exposed to mesoporous silica possibly due to thrombosis [58]. Our next step for assessing toxicity study will include a study of clearance, haematology, serum chemistry and histopathology. This further provides clear evidence for the complex, intrinsically multidimensional nature of toxic interactions between zeolites and biological systems.

## 4. Materials and Methods

### 4.1. Preparation of Zeolite Particles

#### 4.1.1. Materials

The following chemicals from Sigma-Aldrich (St. Louis, MO, USA) were used as reagents for zeolite synthesis: LUDOX AS-40 colloidal silica (40 wt% suspension in water), tetraethylorthosilicate (TEOS, 98%), potassium hydroxide (85% pellets), sodium hydroxide (98%), aluminum sulfate hydrate (98%), aluminum isopropoxide (98%), 1-butanol (ACS reagent, ≥99.4%), and poly diallyldimethylammonium chloride (PDDAC, MW 150K, 20%). Sodium aluminate (technical grade) and tetrapropylammonium hydroxide (TPAOH, 40%) were purchased from Alfa Aesar. The deionized (DI) water used in all experiments was purified with an Aqua Solutions RODI-C-12A purification system (18.2 MΩ). All reagents were used as received without further purification.

#### 4.1.2. Synthesis of Zeolite L (LTL)

LTL crystals were synthesized according to a reported protocol using solutions with a molar ratio of 1.0Al${}_{2}$O${}_{3}$:20SiO${}_{2}$:10.2K${}_{2}$O:1030H${}_{2}$O [25]. Potassium hydroxide (0.69 g, 0.0104 mol) was first dissolved in DI water (ca. 7.6 g), followed by addition of aluminum sulfate hydrate (0.18 g, 0.00051 mol). This solution was stirred until clear (ca. 5 min). LUDOX AS-40 (1.53 g, 0.0102 mol) was then added dropwise, and the resulting solution was left to stir overnight at room temperature. Rod-shaped crystals (referred to as LTL-R) were prepared with PDDAC, whereas disc-shaped crystals (referred to as LTL-D) were prepared with 1-butanol. Each organic zeolite growth modifier (ZGM) was added in a molar ratio of 1.5ZGM:1.0SiO${}_{2}$ to 10 g of growth solution, yielding mixtures with a pH of 14.4 ± 0.2. The solution was placed in a Teflon-lined stainless steel acid digestion bomb (Parr Instruments) and heated without mixing at autogenous pressure in an oven (ThermoFisher Precision Premium 3050 series gravity oven) for 3 days at 180 ${}^{\circ }$C. The reaction product was isolated as a white powder (ca. 300 mg) by vacuum filtration using a 0.4-μm membrane (47 mm Whatman nuclepore polycarbonate track-etched membrane) with repeated DI water washings. For the preparation of microscopy samples, a small amount of powder was redispersed in DI water and shaken vigorously. An aliquot of this solution was then placed on a glass slide and dried overnight. All samples for microscopy studies were prepared via transfer of crystals from the glass slide to carbon tape on the sample holder.

#### 4.1.3. Synthesis of ZSM-5 (MFI-S)

Growth solutions were prepared according to a reported protocol with a molar composition of 6.0TPAOH:0.1Na${}_{2}$O:25SiO${}_{2}$:0.25Al${}_{2}$O${}_{3}$:480H${}_{2}$O:100EtOH [59]. We first added TEOS dropwise to a solution of TPAOH, NaOH, and DI water (25 mL total volume). This solution was stirred overnight at room temperature. Aluminum isopropoxide was added and the mixture was aged for an additional 24 h at room temperature with continuous stirring. The solution was then placed in an acid digestion bomb and was heated in an oven at 100 ${}^{\circ }$C for 60 h. The crystalline product was isolated from the supernatant by the same centrifugation/washing cycles. The water was decanted, leaving behind a gel containing the ZSM-5 crystals that was directly added to a solution with composition 10TEOS:14TPAOH:9500H${}_{2}$O that was prepared by adding an appropriate amount of TEOS (dropwise) to a solution containing TPAOH and DI water (25 mL total volume). The solution was stirred at room temperature overnight prior to the addition of ZSM-5 crystals and heating at 170 ${}^{\circ }$C for 12 days. The spheroidal crystals extracted from this process are referred to herein as MFI-S.

#### 4.1.4. Synthesis of Faujasite (FAU-S)

Zeolite growth solutions were prepared according to a reported protocol using the molar composition 7.33Si:1.83Al:11NaOH:190H${}_{2}$O by first mixing sodium aluminate and sodium hydroxide in DI water [2]. These solutions were stirred until well-mixed, and then LUDOX AS-40 was added as the silica source. Solutions were stirred for 24 h prior to being placed in a Teflon liner, which was loaded into a stainless-steel autoclave and heated in an oven at 65 ${}^{\circ }$C for 7 days. The solid crystals were recovered by three cycles of centrifugation and washing with DI water. Centrifugation was performed using a Beckman Coulter Avanti J-E at 5 ${}^{\circ }$C and 13,000 rpm for 45 min. The gel product was dried in air at room temperature overnight prior to analysis. The resultant spheroidal crystals are referred to herein as FAU-S.

### 4.2. Dispersion and Characterization of Materials

The zeolites were dispersed in DI water, Dulbecco’s phosphate-buffered saline (DPBS) or culture medium. In a typical batch reaction, an aqueous solution (20 mL) containing dispersed zeolites in a bottle was kept on a shaker at appropriate temperature. Based on success of homogeneous dispersion studies using physical mixing and sonication, stock solutions were prepared either in PBS or DI water. From this stock solution, differential concentrations were prepared in cell growth medium. It was noted that turbidity increased with increasing concentration of zeolites. The turbidity intensified significantly at the 250 μg/mL for all zeolite solutions. After incubation for 5 min, the morphology and size of the zeolite crystals were observed by scanning electron microscopy (SEM), which was conducted at the Methodist Hospital Research Institute (Houston, TX, USA) in the Department of Nanomedicine SEM Core using a Nova NanoSEM 230 instrument with ultra-high-resolution FESEM (operated at 15 kV and a 5 mm working distance). All zeolite samples were coated with ca. 15 nm layer of Pt metal prior to imaging to reduce the effects of charging. The pH of growth solutions was measured with a Thermo Scientific Orion 3 Star meter. The crystalline structure of each zeolite sample was verified using X-ray diffraction (XRD) where powder patterns were collected on a Siemens D5000 X-ray diffractometer with Cu Kα radiation (40 kV, 30 mA) in the 2θ range of 7–50 ${}^{\circ }$ and a scanning rate of 1 ${}^{\circ }$/min. The crystal phase was indexed using simulated patterns, which we obtained from the International Zeolite Association Structure Database (H. Gies and H. van Koningsveld 2017). The elemental composition of zeolite samples was determined by inductively coupled plasma atomic emission spectroscopy (ICP-AES) at Galbraith Laboratories (Knoxville, TN, USA).

### 4.3. Zeta Potential Measurements

The zeta potential of zeolite particles was measured using the Malvern Zetasizer Nano ZS (Malvern Instruments, Malvern, UK). Zeolite particles were suspended in DI water (resistivity > 18.2 MΩ·cm; Direct-Q, Millipore, Burlington, USA), DPBS (WelGENE Inc., Daegu, Korea), and Eagle’s minimum essential medium (EMEM, American type culture collection (ATCC), Rockville, MD, USA) culture medium with or without 10% fetal bovine serum and 100 U/mL penicillin-streptomycin (S 001-07 and LS 202-01, both from WelGENE Inc., Gyeongsan-si, Korea). Then, the samples were sonicated in an ultrasonic bath (POWER SONIC 410, Hwashin, Korea) for 5 min. The condition of all zeolites after sonification was carefully checked by observing SEM Images to confirm all samples were undamaged.

### 4.4. Cell Cultures

The IMR-90 lung fibroblast cells were obtained from American Type Culture Collection (ATCC CCL-186, Rockville, MD, USA) and maintained in a humidified atmosphere at 37 ${}^{\circ }$C and 5% CO${}_{2}$. The IMR-90 cells were cultured in EMEM (ATCC 30-2003, Rockville, MD, USA), containing 10% (*v*/*v*) FBS and 100 U/mL penicillin-streptomycin (S 001-07 and LS 202-01, both from WelGENE Inc., Gyeongsan-si, Korea). The cells were split sub-confluent cultures (70%), seeding at 10,000 cells/cm${}^{2}$ using 0.25% trypsin; 5% CO${}_{2}$; 37 ${}^{\circ }$C. The experiment is approved by institutional review board (SC19ZNSE0016).

### 4.5. Real-Time Cell Monitoring

An xCELLigence system (Roche Applied Science) was used to measure the cellular response upon zeolite particle treatment. Cell attachment, spreading and proliferation were continuously monitored every 15 min. The concentration of zeolite particles is based on a previous study [32]. The cells were seeded in E-plate 16 (ACEA Biosciences, Inc., San Diego, CA, USA) with a density of 1 × 10^4^ cells/well and cultured for 24 h in a humidified atmosphere at 37 ${}^{\circ }$C and 5% CO${}_{2}$. Then, the four types of zeolites (LTL-R, LTL-D, MFI-S, and FAU-S), suspended in media were added to the respective wells at different concentrations (10, 50, and 100 μg/mL). The zeolite particles without cells were set as the control, and the cell culture media was replaced every 2–3 days. This experiment was performed in quadruplet and continued for several days. The impedance value of each well was automatically monitored and expressed as a CI (cell index) value. Data for cell adherence were normalized at the time of the zeolite treatment. Normalized CI is calculated by dividing CI at particular time points by the CI at the time of interest [53].

### 4.6. WST Cell Viability Assay

The effect of zeolites on the viability of IMR-90 cells were examined using water soluble tetrazolium salt (WST) assay. The IMR-90 cells (5 × 10^3^ cells/well) were seeded in a 96-well plate and allowed to stabilize for 24 h at 37 ${}^{\circ }$C in a 5% CO${}_{2}$ incubator. Then, the cells were exposed to different concentrations of zeolite particles (10, 50, and 100 μg/mL) for 24 h. The cell viability was measured by adding 10 μL/well of metabolic cell proliferation reagent EZ-Cytox (EZ-1000, Daeil Labservice, Seoul, Korea). After 2 h of culturing, the cell viability was determined as a percentage by comparing the absorbance at 450 nm for the tested cells and untreated control cells using an ELISA reader (Power wave XS, BioTek), and calculated against a background control (media). The experiment was performed in triplicate.

### 4.7. Cytotoxicity Assay

The cytotoxic effect of zeolite particles on IMR-90 lung fibroblast cells was analyzed using an LDH cytotoxicity detection kit (04744926001, Roche Applied Science). The cells were plated in quadruplicate at a density of 5 × 10^3^ cells/well in a 96-well plate and grown for 24 h at 37 ${}^{\circ }$C. Various concentrations of zeolite particles (0, 10, 50, 100, 250 μg/mL) were added to each well and incubated for 72 h. Finally, each well was treated with the LDH reagents according to the manufacturer’s instructions and measured using a microplate reader (Power wave XS, BioTek) at 492 and 690 nm. The experiment was carried out in triplicate. The percentage cytotoxicity was calculated as follows: [(experimental value − low control)/(high control − low control)] × 100%. Mitochondrial function was evaluated spectrophotometrically by measuring the degree of mitochondrial reduction of the tetrazolium salt 3-(4,5-dimethylthiazol-2-yl)-2,5-diphenyltet- razolium bromide (MTT) to (aqueous insoluble product) formazan by succinic dehydrogenase [60], which was modified as described previously [61].

### 4.8. Mitotracker

IMR-90 cells were plated in 96 well plate and treated with 10, 50, 100, 250 μg/L LTL-R, LTL-D, MFI-S and FAU-S for 72 h. After incubation for 30 min, the cells were treated with 200 nmol/L MitoTracker Red (Molecular Probes/Invitrogen, Carlsbad, CA, USA) for 25 min at 37 ${}^{\circ }$C and 5% CO${}_{2}$, fixed using the Image-iT fixation/permeabilization kit (Molecular Probes), washed three times with PBS, and non-specific sites were blocked with PBS containing 2 g/L bovine serum albumin for 60 min at 25 ${}^{\circ }$C. Cells were then stained with 200 μL of 3× Dulbecco’s PBS (DPBS) containing 5 μL of Alexa Fluor 488 phalloidin (200 units/mL in methanol; Molecular Probes) for 20 min and washed with DPBS; nuclei were counterstained with NucBlue Fixed Cell ReadyProbes Reagent (R37606; Molecular Probes/Invitrogen). The slides were covered with coverslips and cells were observed and photographed under a confocal laser scanning microscope (LSM 710; Carl Zeiss, Jena, Germany).

### 4.9. Mitochondrial Membrane Potential

Cells were seeded in a 24-well plate at 5 × 10${}^{4}$ cells/well and incubated in a CO${}_{2}$ exposed to different concentration of each zeolites for 72 h. Mitochondrial function was determined by uptake of rhodamine 123 (Molecular Probes, Inc., Eugene, OR, USA) as previously described [62]. Cells were exposed to different concentrations of zeolites for 72 h. After 72 h-exposure, cells were incubated with rhodamine 123 for 30 min in a 96-well plate then cells were washed with PBS. The fluorescence was determined at excitation wavelength 485 nm and emission wavelength 530 nm. Control cells cultured in zeolite-free media were run in parallel to treatment groups. The fluorescence intensity value of control cells (zeolite-free medium at 0 h) was taken as 100% and then calculated as the percentage of reduction of fluorescence in zeolite-exposed cells. Image Examiner software (Carl Zeiss).

### 4.10. Quantification of Reduced (GSH) and Oxidized Glutathione (GSSG)

Qauantities of GSH, oxidized glutathione (GSSG), and GSH/GSSG were determined by luminescence using the GSH/GSSG-Glo assay kit (Promega, Korea). The assay was assessed according to the manufacturer’s instructions. Briefly, IMR-90 cells were treated with 72 h MTT EC${}_{50}$ of the corresponding zeolites. After treatment, cells were centrifuged and washed with cold 1× PBS. The pellet was resuspended with 200–500 μL ice-cold 0.5% metaphosphoric acid (MPA), the cells were again centrifuged at 1200 rpm for 5 min at 4 ${}^{\circ }$C and the supernatant was collected. Then, 25 μL 1× glutathione reductase and 25 μL 1× NADPH were added in 96-well plate and after that μ100 L of the samples were added. Finally, 50 μL 1× chromogen was added and mixed briefly. Immediately, the absorbance was recorded at 405 nm at 2 min intervals for 10 min. Each plate was read using a microplate reader (Biotek, Winooski, VT, USA) luminescence. The total glutathione content was determined by comparison with the predetermined glutathione standard curve. The results were expressed as percentage of total glutathione (GSSG/GSH) content.

### 4.11. Transmission Electron Microscopy

The IMR-90 cells were seeded in a 6-well plate and incubated with or without 50 μg/mL zeolite particles in a humidified atmosphere at 37 ${}^{\circ }$C and 5% CO${}_{2}$ for 72 h. The cells were centrifuged and prefixed with 2% glutaraldehyde in 0.1 M phosphate buffer for 2 h at room temperature. The samples were washed and fixed in 1% osmium tetroxide for 2 h. Then, the cells were dehydrated in a graded series of ethanol (40 to 100%) before being embedded in epoxy resin. Ultrathin sections of 80 nm were cut by sectioning using an ultramicrotome with a diamond knife (Leica EM UC6, Vienna, Austria). The sections were prepared on EM grids and counterstained using uranyl acetate and lead citrate to examine under a JEOL 1200EX electron microscope (JEOL, Tokyo, Japan).

### 4.12. RNA Isolation, PCR Arrays, and qRT-PCR Assays

To collect RNA from cultured cells, Cultured IMR-90 cells were incubated for 72 h with EMEM medium supplemented with 10% FBS and 100 U/mL penicillin-streptomycin, containing 50 μg/mL each of absence of zeolite (control), LTL-R, LTL-D, MFI-S and FAU-S. The RT${}^{2}$ profiler PCR array Human Stress and toxicity PathFinder (PAHS-003Z, Qiagen, Manchester, UK) was used to analyze the expression of fibrosis related genes in IMR-90 cells. Total RNA was used to synthesize first strand cDNA using the RT${}^{2}$ First Strand Kit (330404, Qiagen). The cDNA was diluted to 111 μL by adding RNase-free water and stored at −20 ${}^{\circ }$C until use. The 12 μL of cDNA was mized with RT${}^{2}$-SyBR Green qPCR Master added to a (330520, Qiagen) and 25 μL of mixture was added RT${}^{2}$ Profiler${}^{\mathrm{TM}}$ PCR array plate. Each plate contains predesigned gene-specific primers including a panel of 84 genes related to human fibrosis and five separate housekeeping genes. The data of two arrays were normalized by using five house-keeping genes (beta-2-microglobulin [B2M], hypoxanthine phosphoribosyltransferase 1 [HPRT1], ribosomal protein L12a [RPLP0], glyceraldehyde-3-phosphate dehydrogenase [GAPDH], and beta-actin [ACTB]) included on the same assay plate. The fold-change was calculated based on the expression of each gene in the asbestos-treated groups versus that in the untreated group. ΔΔCT value was calculated as the difference between ΔCT of treatment group and that of control. A 2-fold cutoff threshold was used to define significantly up-regulated or down-regulated genes. Resulting data were uploaded to the Qiagen web portal (http://www.qiagen.com (accessed on 5 May 2021), normalized to averaged reference transcripts All experiments were performed in triplicate and using the Thermal Cycler Dice Real Time System TP800 2.10B (Takara Bio, Tokyo, Japan).

### 4.13. Statistics

All results were analyzed using the GraphPad Prism version 9.0.0 (GraphPad software, San Diego, CA, USA). All data were representative of experiments done in at least triplicate and were represented as mean ± standard deviation (SD). The statistical significance of differences was analyzed using a t-test and one-way analysis of variance (ANOVA) followed by Dunnett’s method for multiple comparison. *P*-values less than 0.05 were considered statistically significant.

## 5. Conclusions

In conclusion, it is imperative to understand the design, development, and use of zeolites with respect to properties such as morphology and composition to ensure that these materials are optimal with respect to their corresponding biological and environmental effects. In addition, those who are engaged in occupations that use zeolite particles cannot be excluded from the possibility of exposure, which has the potential to cause cytotoxic effects and burden of lung disease with extended exposure, thus highlighting the need for awareness to take precautions that prevent exposure.

## Figures and Tables

**Table 1:** Physicochemical properties of zeolite particles.

	LTL-R	LTL-D	MFI-S	FAU-S
Si/Al ${}^{a}$	3	3	50	1.5
Zeta potential (mV)				
DI water ${}^{b}$	−52 ± 1	−68 ± 4	−39.3 ± 0.8	−43.4 ± 0.5
DPBS ${}^{c}$	−28.9 ± 0.2	−22.6 ± 0.8	−29 ± 1	−28.4 ± 0.2
EMEM-N ${}^{d}$	−28 ± 2	−14.1 ± 0.4	−27 ± 1	−21 ± 1
EMEM-F *^e^*	−10.5 ± 0.4	−9.3 ± 0.5	−10.3 ± 0.2	−11.8 ± 0.6

*^a^* Si/Al, silicon-to aluminum ratio. *^b^* Millipore-Q, pH 6.69. *^c^* Dulbecco’s phosphate-buffered saline (Welgene), pH 7.3. *^d^* Eagle’s minimum essential medium (ATCC), pH 7.1. *^e^* Eagle’s minimum essential medium (ATCC) supplemented with 10% FBS and 1% penicillin-streptomycin, pH 7.2.

**Table 2:** Calculated EC${}_{50}$ values represent effective concentration of zeolites that increases LDH leakage to 50% or decreases MTT reduction by 50%.

Zeolite	LDH EC${}_{50}$, μg/mL	MTT EC${}_{50}$, μg/mL
LTL-R	148.8 ± 22.3	161.0 ± 18.6
LTL-D	214.9 ± 55.6	225.5 ± 28.3
MFI-S	144.5 ± 24.2	148.7 ± 17.7
FAU-S	57.0 ± 13.2	57.8 ± 10.6

Values were calculated by using linear statistical regression analysis.

**Table 3:** Gene expression of TNF related component in zeolite exposed lung fibroblast using qRT-PCR.

TNF Related Genes Mean Fold Change ± SD	LTL-R	LTL-D	MFI-S	FAU-S
CD40LG	1.12 ± 0.23	1.19 ± 0.32	1.56 ± 0.14	6.33 ± 0.55
	*p* = 0.4679	*p* = 0.3901	*p* = 0.006	*p* < 0.001
TNF-alpha	0.97 ± 0.33	1.42 ± 0.34	1.83 ± 0.32	4.32 ± 0.33
	*p* = 0.888	*p* = 0.075	*p* = 0.012	*p* < 0.001

Cells were exposed to 50 μg/mL of each zeolite for 72 h. qRT-PCR was used to confirm the relative gene expressionchanges detected in PCR arrays. Mean fold changes in gene expression were calculated relative to untreated cells on 2 independent experiments. Fold changes are compared to control by Student’s *t* test. A *p*-value < 0.05 is considered statistically significant.

## Data Availability

The datasets generated during and/or analysed during the current study are available from the corresponding author on reasonable request.

## Sample Availability

Samples of the zeolites are available from the authors.
